# Ceftriaxone and cefazolin attenuate the cue-primed reinstatement of alcohol-seeking

**DOI:** 10.3389/fphar.2015.00044

**Published:** 2015-03-10

**Authors:** Ana Weiland, Steven Garcia, Lori A. Knackstedt

**Affiliations:** ^1^Department of Neuroscience, Medical University of South CarolinaCharleston, SC, USA; ^2^Department of Psychology, University of FloridaGainesville, FL, USA

**Keywords:** glutamate, alcohol, GABA, cocaine, addiction, relapse, *N*-acetylcysteine, ceftriaxone

## Abstract

Alcohol consumption and the reinstatement of alcohol-seeking rely on glutamate and GABA transmission. Modulating these neurotransmitters may be a viable treatment strategy to prevent alcohol relapse. *N*-acetylcysteine (NAC) and the antibiotic ceftriaxone (CEF) alter the glial reuptake and release of glutamate while the antibiotic cefazolin (CEFAZ) modulates GABA signaling without affecting glutamate. Here, we used the extinction-reinstatement model of relapse to test the ability of these compounds to attenuate the reinstatement of alcohol-seeking. Male Sprague-Dawley rats were trained to self-administer 20% (v/v) alcohol in the home cage using an intermittent schedule (24 h on, 24 h off) for 12 sessions. Subsequently, animals self-administered alcohol during daily 45-min operant sessions for 26 sessions, followed by extinction training. We tested whether chronic administration of NAC, CEF, or CEFAZ attenuated the cue-primed reinstatement of alcohol-seeking. CEF and CEFAZ attenuated cue-primed reinstatement of alcohol-seeking while NAC had no effect. We subsequently investigated whether CEF and CEFAZ alter the self-administration of sucrose and chow pellets and if CEFAZ attenuates the reinstatement of cocaine-seeking. The operant self-administration of regular chow and sucrose was not altered by either CEF or CEFAZ. CEFAZ had no effect on cocaine reinstatement, a behavior that has been strongly tied to altered glutamate homeostasis in the nucleus accumbens. Thus the ability of CEFAZ to attenuate alcohol reinstatement likely does not involve the glial modulation of glutamate levels. The dampening of GABA transmission may be a common mechanism of action of cefazolin and ceftriaxone.

## Introduction

Alcoholism or alcohol dependence is a chronic, progressive disease which results from an inability to regulate drug-seeking behavior. Approximately 17 million Americans are dependent on alcohol (NIH, 2006), resulting in significant legal and medical costs to society totaling an estimated $184 billion per year (Kenna et al., 2004). At present, only disulfiram, naltrexone, and acamprosate are approved by the Food and Drug Administration to treat alcoholism. However, the ability of these medications to reduce the risk of relapse is modest (Heilig and Egli, 2006; Soyka and Roesner, 2006) and thus there is a need to develop more targeted pharmacological treatments for alcohol addiction.

Animal models of relapse permit the identification of the underlying neurobiology of alcohol relapse in humans and thus the targeted development of pharmacotherapies. Relapse is modeled in animals with the reinstatement paradigm, where animals are trained to self-administer drug in an operant chamber. The drug-seeking response is extinguished and reinstated with one of the stimuli known to cause relapse in humans such as stress, cues associated with drug delivery, or the drug itself (Epstein et al., 2006). It has proven challenging to get laboratory rats to voluntarily consume doses of alcohol relevant to human alcohol addiction. Various procedures have been attempted to increase oral consumption of alcohol in rodents, such as sucrose fading (Samson, 1986), food and water-deprivation (Meisch and Thompson, 1972), alcohol vapor exposure (Walker and Koob, 2007; Walker et al., 2008), and the use of rat strains selectively bred for high preference to alcohol (Samson et al., 1998; Vacca et al., 2002). Since its introduction in the 1980s, sucrose fading was largely adopted as the chief method of inducing rodents to self-administer alcohol in an operant setting (Samson, 1986). While sucrose fading yields high alcohol consumption in the presence of sucrose, drinking drops sharply when the sweetener is removed (Samson, 1986; Koob and Weiss, 1990; Samson et al., 1999; Carrillo et al., 2008). Additionally, sucrose has addictive properties itself (Colantuoni et al., 2002; Avena et al., 2008), possibly contributing to the motivation to consume sweetened alcohol. One method of inducing high amounts of unsweetened alcohol consumption in rodents is the intermittent access to alcohol (IAA) procedure in which rodents are provided 24 h access to alcohol followed by a 24 h period with no access (Wayner and Greenberg, 1972; Wise, 1973; Pinel and Huang, 1976). Simms et al. (2008) recently resurrected this model and induced levels of drinking (20% alcohol v/v) in the 5–8 g/kg/day range in Wistar and Long-Evans strains. Furthermore, Simms et al. (2010) adapted the IAA paradigm to an operant self-administration model and engendered high levels of alcohol-seeking in the operant chamber after using 14 h operant sessions on intermittent days (MWF), followed by shorter (30–45 min) daily sessions in the operant chamber. Here, we modified this method to eliminate the use of 14 h operant sessions and trained animals in the operant chamber only during daily 45-min sessions for 26 sessions (MTWRF).

We then sought to test the ability of translational compounds which modulate glutamate and/or GABA transmission to attenuate the cue-primed reinstatement of alcohol-seeking. Glutamate transmission in general and specifically in the nucleus accumbens (NAs) and amygdala is strongly implicated in cue-induced alcohol reinstatement. During the cue-primed reinstatement of extinguished operant alcohol-seeking, synaptic glutamate release increases in the NA and the amygdala (Gass et al., 2011). Thus, it follows that both systemic and intra-NA or intra-amygdala infusion of antagonists to post-synaptic glutamate receptors attenuate alcohol reinstatement (e.g., Backstrom and Hyytia, 2004; Backstrom et al., 2004; Sanchis-Segura et al., 2006; Sinclair et al., 2012).

The pharmacological manipulations which prevent relapse to alcohol-seeking also prevent the reinstatement of cocaine-seeking (for review see Kalivas et al., 2009; Knackstedt and Kalivas, 2009). Following cocaine self-administration and withdrawal, evidence exists for altered glutamate homeostasis, defined as the homeostatic regulation of synaptic and extrasynaptic glutamate levels and signaling. Glutamate homeostasis in the accumbens is complex and regulated by multiple mechanisms, including by the major glutamate transporter GLT-1 and by system x_c_-. System x_c_- exchanges extracellular cystine for intracellular glutamate, thereby contributing the majority of basal extrasynaptic glutamate in the accumbens (Baker et al., 2002). The catalytic subunit of system x_c_- is the protein xCT and the hallmarks of altered glutamate homeostasis after cocaine include a reduction in expression and function of both GLT-1 and xCT/system x_c_- (Knackstedt et al., 2010; Trantham-Davidson et al., 2012). Based on the widespread involvement of various types of glutamate receptors in alcohol relapse, it is likely that glutamate homeostasis is altered following alcohol consumption as well.

Modulation of GABA transmission also impacts alcohol consumption and reinstatement. GABA_A_ antagonists and inverse agonists decrease alcohol consumption (see review by McBride and Li, 1998) and operant self-administration of alcohol (Samson et al., 1987).The GABA_B_ (autoreceptor) agonist baclofen decreases self-administration in non-dependent rats (Janak and Michael Gill, 2003). Conversely, allopregnanolone, an endogenous neurosteroid which has also been shown to be a potent positive modulator of GABA_A_ receptors, dose-dependently induces the reinstatement of alcohol-seeking (Nie and Janak, 2003; Finn et al., 2008).

We hypothesized that glutamate homeostasis is altered post-alcohol administration, and restoring homeostasis would reverse alcohol-induced plasticity and thereby attenuate the reinstatement of alcohol-seeking. It is possible to restore glutamate homeostasis and reverse cocaine-induced pathologies in synaptic plasticity following with *N*-acetylcysteine (NAC; Moussawi et al., 2009, 2011) or ceftriaxone (CEF; Trantham-Davidson et al., 2012) and here we tested their ability to attenuate cue-primed reinstatement of alcohol-seeking. NAC serves as a cystine prodrug that drives system x_c_- to export more glutamate while CEF is an antibiotic that upregulates the expression of both GLT-1 (Lee et al., 2008) and xCT (Lewerenz et al., 2009) at the transcriptional level. We also hypothesized the antibiotic cefazolin (CEFAZ), which has been demonstrated to inhibit GABA_A_ transmission (Yamazaki et al., 2002; Sugimoto et al., 2003), would attenuate cue-primed alcohol reinstatement. In order to investigate a possible effect of CEFAZ on glutamate homeostasis, we tested its ability to attenuate the reinstatement of cocaine-seeking, a behavior that is dependent on altered glutamate release, clearance and basal levels (for review see Knackstedt and Kalivas, 2009).

## Materials and Methods

### Animals and Housing

Adult male Sprague-Dawley rats (*n* = 84), weighing 250–275 g upon arrival were individually housed in ventilated Plexiglas cages in a climate-controlled room on a 12-h reverse light/dark cycle (lights off at 9 AM) and given at least 1 week to acclimate to the individual housing conditions and handling. Rats were provided ad libitum water throughout the experiment and were food-deprived to ∼85% of free-feeding weight with a daily allowance of 20 g of food. Food-restriction generally increases responding without altering reinstatement relative to non-restricted controls (Bongiovanni and See, 2008). Experiment 1 was conducted at the Medical University of South Carolina and Experiments 2–4 were conducted at the University of Florida. All procedures were pre-approved by the Medical University of South Carolina and University of Florida Institutional Animal Care and Use Committees and were in accordance with National Institutes of Health *Guide for the Care and Use of Laboratory Animals*.

### Operant Chambers and Training

Prior to beginning operant self-administration (Experiments 1–4), animals underwent a single, overnight food-training session in the operant chambers where presses on the active lever resulted in the delivery of food pellets. Operant chambers (Med Associates) were equipped with two retractable levers, stimulus lights, and tone generators. On the day following food training, animals were trained to self-administer alcohol, cocaine, or food pellets in the operant chamber. For the food self-administration study (Experiment 3), presses on the active lever delivered food pellets. For drug self-administration studies, presses on the active lever activated a pump which dispensed drug. For alcohol studies (Experiments 1 and 2), 0.1 mL alcohol (20% v/v) was dispensed into a dipper tray; infrared sensors verified head entry into the trough area to consume the alcohol. For the cocaine study (Experiment 4), 0.25 mg cocaine HCl (kindly provided by NIDA) in 0.1 mL 0.9% saline was delivered per infusion. For both cocaine and alcohol studies, drug delivery was accompanied by the presentation of discrete cues (a light and 2900 Hz tone) and for cocaine self-administration, was followed by a timeout (20 s) during which time presses on the active lever did not yield drug. Inactive lever presses had no consequences, but were recorded. For alcohol self-administration, the trough was blotted with a tissue at the end of the session to verify that all alcohol was consumed. Rats who habitually did not consume alcohol despite lever presses were eliminated from the experiment (*n* = 3 for Experiment 1; *n* = 2 for Experiment 2). Additionally, three rats that were in ill-health were removed from Experiments 1 and 2. For both alcohol and cocaine self-administration studies, animals self-administered drug for a pre-determined number of days in the operant boxes (26 for alcohol; 12 for cocaine) regardless of whether drug intake was stable or not. This method was chosen because we wanted to prevent animals from self-administering drug for varying amounts of time which could lead to a wide range of drug intake. The amount of cocaine consumed during self-administration has been correlated with later drug-seeking during reinstatement tests (e.g., Deroche et al., 1999; Baker et al., 2001).

### Alcohol Self-Administration, Extinction Training, and Reinstatement Testing

All rats that later self-administered alcohol in the operant chambers were first trained to drink with the IAA (e.g., Simms et al., 2008). This paradigm provides rats with 24-h access to unsweetened 20% alcohol on alternating days (3 days/weeks) without water-deprivation. Animals experienced 12 sessions (over 4 weeks) of the intermittent-access drinking paradigm.

Following exposure to alcohol using the IAA, animals were trained to self-administer 20% alcohol (v/v) using daily 45 min training sessions on an FR-1 schedule of reinforcement for 6 days followed by an FR-3 schedule for 20 daily sessions. This procedure followed that of Simms et al. (2010), with the exception of using 45 min FR-3 operant sessions in place of 30 min sessions and replacing the 12 overnight operant sessions with 12 IAA sessions. Extinction training began following the 20th FR-3 session. During extinction training, presses on the previously active lever no longer provided drug and cue presentation. Extinction training lasted a minimum of 10 sessions and until animals reached the criteria of less than 20 lever responses per session. Once this criteria was reached, animals underwent a 45 min cue-primed reinstatement test during which presses on the previously active lever once again yielded presentation of discrete cues that had been paired with alcohol delivery (stimulus light, tone and the sound of the syringe pump activating). In addition, 2 mL of 20% EtOH was sprinkled on the bedding of the operant chamber to provide an olfactory cue.

#### Experiment 1

**FIGURE 1:**
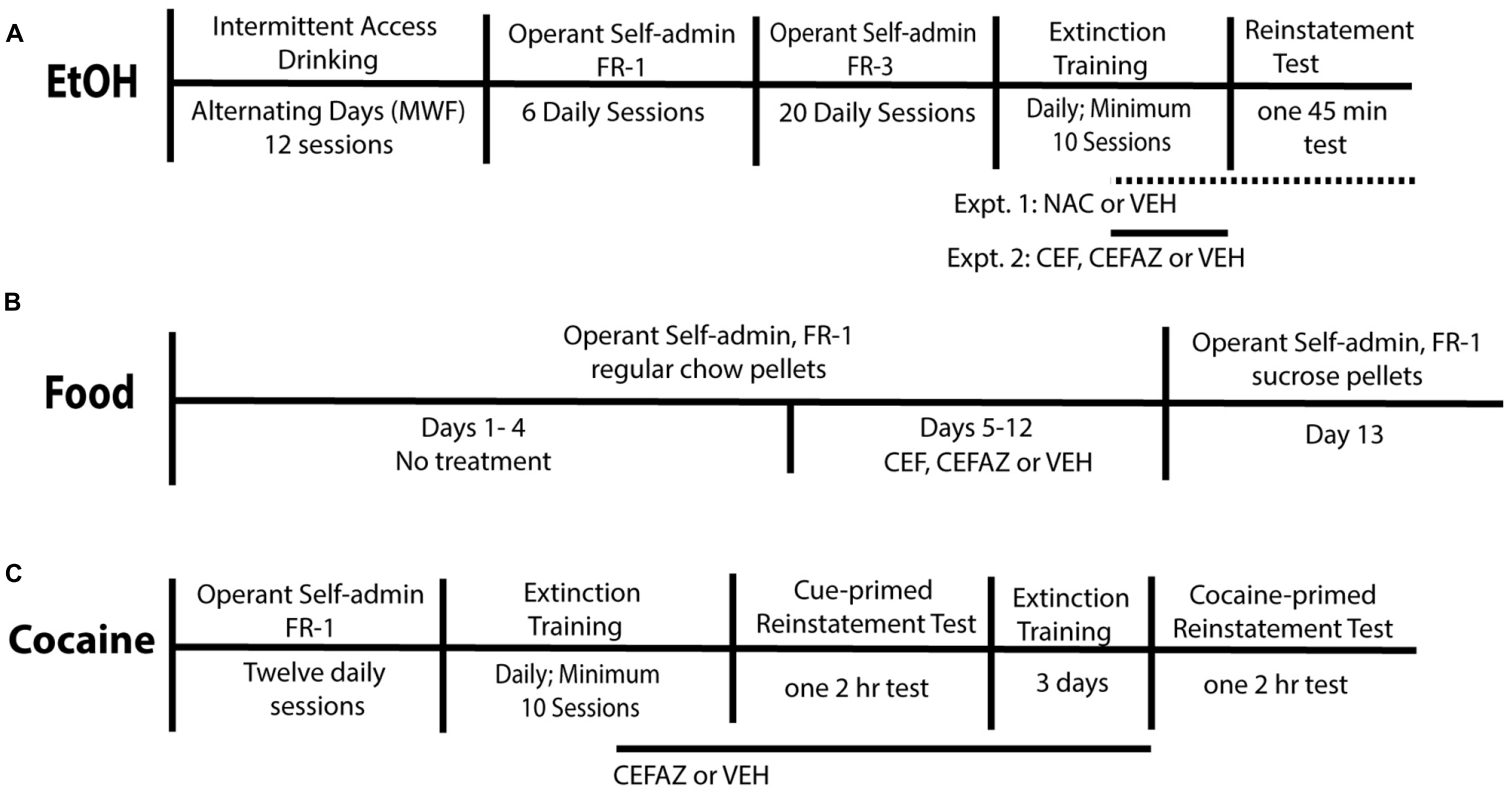
Experimental Methods. **(A)** Timeline of the experimental procedure for Experiments 1 and 2. All animals were trained to self-administer food pellets immediately prior to beginning operant alcohol self-administration. VEH, NAC, CEF, and CEFAZ treatment commenced on the last 6–8 days of extinction training. For the NAC experiment, animals were also injected 2 h prior to the test. **(B)** Timeline of the experimental procedure for Experiment 3. All animals were trained to self-administer food pellets immediately prior to beginning operant alcohol self-administration. VEH, CEF, and CEFAZ treatment commenced on Day 5 of food self-administration and continued through Day 12. **(C)** Timeline of the procedure for Experiment 4. All animals were trained to self-administer food pellets immediately prior to beginning operant cocaine self-administration. VEH and CEFAZ was administered for the last 6–8 extinction sessions and continued after the cue-primed reinstatement test.

Here, we tested the ability of chronically administered NAC to attenuate the cue-primed reinstatement of alcohol-seeking. *N*-acetylcysteine (Sigma, 30 or 60 mg/kg IP) or vehicle (VEH; saline 0.3 mL IP) was administered 2 h prior to the last 6–8 extinction sessions and prior to the reinstatement test (see **Figure 1A**). The dose and timing of injections was based on previous studies demonstrating the ability of 60 mg/kg NAC to restore basal glutamate levels after cocaine and attenuate cocaine reinstatement when given both acutely prior to testing (Baker et al., 2003) and chronically during extinction training (Amen et al., 2011) while a lower dose (33 mg/kg) only partially inhibits cocaine-seeking (Moussawi et al., 2009). NAC works acutely, within 2 h of administration, to increase basal glutamate by serving as an exogenous source of cysteine, which drives system x_c_- to export more glutamate (Baker et al., 2003), but also alters glutamate homeostasis when administered daily for 5 or more days (Knackstedt et al., 2010; Amen et al., 2011). After reaching extinction criteria, the responding of the NAC-treated animals during a cue- primed reinstatement trial was compared with VEH-treated animals. Animals were assigned to receive NAC or VEH treatment in a counterbalanced manner to ensure no group differences in drug self-administration. One animal was excluded from reinstatement testing (and all data analysis) due to a failure to reach extinction criteria.

#### Experiment 2

This experiment was carried out in an identical manner to Experiment 1, with the exception that CEF (Hospira, 200 mg/kg IP), CEFAZ (Sigma, 100 mg/kg IP), or VEH (0.9% saline) were administered immediately following the last 6–8 extinction sessions, but not on test day (see **Figure 1A**). After reaching extinction criteria, the responding of the CEF and CEFAZ- treated animals during a cue- primed reinstatement trial was compared with VEH -treated animals. The dose and timing of CEF injections was based on previously published reports showing that 200 mg/kg effectively attenuates cocaine reinstatement and restores glutamate homeostasis when given chronically *after* extinction sessions for at least 5 days (Knackstedt et al., 2010; Trantham-Davidson et al., 2012). The dose of CEFAZ was based on allometric calculations from the maximum human dose recommended by the manufacturer to the equivalent rat dose of 100 mg/kg maximum and was given after extinction sessions akin to the administration of CEF.

#### Food and Sucrose Self-Administration (Experiment 3)

Animals self-administered standard chow pellets (45 mg; Bio-serv) in the operant chamber using an FR-1 schedule of reinforcement for 2 h/day for 12 days. On the 13th day, sucrose pellets (45 mg; Bio-serv) replaced the standard chow pellets. Animals received CEF, CEFAZ, or VEH in an identical manner as described for Experiment 2, with the exception that injections began after the operant session on Day 5 of food self-administration (see **Figure 1B**).

#### Cocaine Self-Administration, Extinction, and Reinstatement (Experiment 4)

For the implantation of catheters, rats were anesthetized with ketamine HCl (87.5 mg/kg, IM) and xylazine (5 mg/kg, IM). Ketorolac (3 mg/kg, IP) was administered before surgery to provide analgesia. Catheter construction and surgical implantation is described in detail elsewhere (Knackstedt et al., 2014). Rats recovered 6 days after surgery prior to beginning self-administration training. Cocaine self-administration was conducted using an FR-1 schedule; sessions lasted 2 h/day and are described in more detail above. Self-administration continued until subjects had attained 12 days with a minimum of 10 cocaine infusions and was followed by extinction training. Animals were either treated with CEFAZ or VEH in manner identical to that in Experiment 2, immediately following the last 6–8 extinction sessions (see **Figure 1C**). Once extinction criteria was attained, animals were tested for cue- and cocaine-primed reinstatement (each test lasting 2 h), with tests separated by 3 days of extinction training. During cue-primed reinstatement, presses on the previously active lever once again yielded the discrete cues paired with cocaine delivery but no drug was delivered. During cocaine-primed reinstatement, animals were injected with 10 mg/kg cocaine (IP) immediately prior to being placed into the operant chamber; presses on the previously active lever had no consequences during this test.

### Data Analysis

The alpha level was set at *p* < 0.05 for all statistical tests, which were conducted using SPSS software. Active lever presses during self-administration and extinction components of the experiment were analyzed with mixed-factorial 2-way analysis of variances (ANOVAs) test, with time as the repeated measure. Reinstatement tests were planned comparisons (extinction vs. test day) and thus were analyzed with paired-sample *t*-tests. Independent-sample *t*-tests were used to compare active lever presses during reinstatement tests between treatment groups. Group differences in food self-administration were examined using one-way ANOVAs. Outliers were defined as values more than two standard deviation +/– the mean and were excluded from further analysis. Groups with outlier values are mentioned in the Results section.

## Results

### The Effects of NAC on Cue-Primed Reinstatement of Alcohol-Seeking (Experiment 1)

**FIGURE 2:**
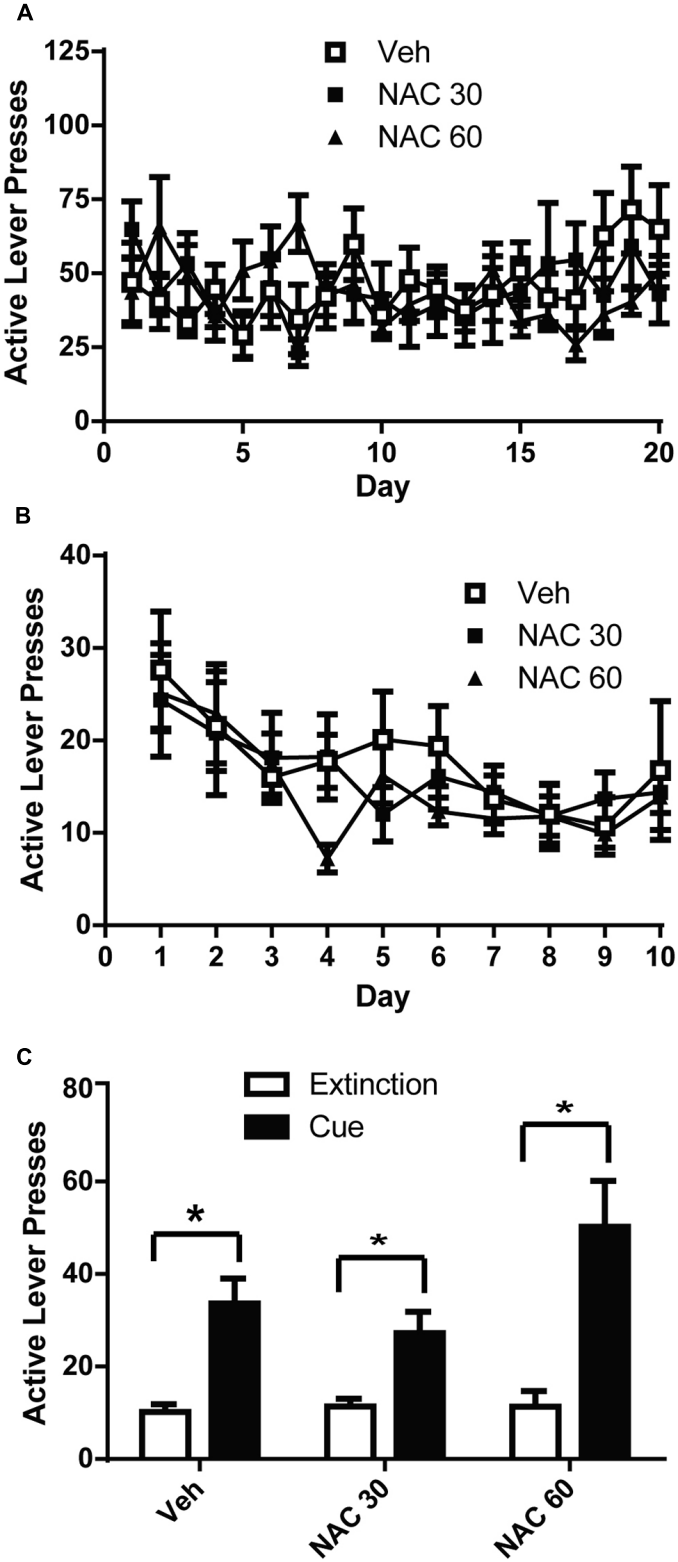
*N*-acetylcysteine did not attenuate cue-primed reinstatement of alcohol-seeking. **(A)** Mean active lever presses during the FR3 portion of the experiment did not differ between groups. **(B)** Mean active lever presses during extinction training did not differ between groups. **(C)** *N*-acetylcysteine did not attenuate cue-primed reinstatement of alcohol-seeking. *p < 0.05 comparing extinction to test.

VEH- and NAC-treated groups did not differ in the amount of alcohol self-administered (**Figure 2A**). A two-way ANOVA with repeated measures on Day was computed on the active lever press data during self-administration and found no significant effect of Day [*F*(19,475) = 1.480, n.s.], or Group [*F*(2,25) = 0.080, n.s.]. There was a significant Day × Group interaction [*F*(19,475) = 2.020, *p* < 0.01]. Similarly, a two-way ANOVA was computed on the lever presses during extinction training (**Figure 2B**) and revealed a significant effect of Day [*F*(9,225) = 6.977, *p* < 0.001], but not Group [*F*(2,25) = 0.240, n.s.]. There was not a significant Day × Group interaction [*F*(18,225) = 0.809, n.s.]. We used paired-sample *t*-tests to analyze the data presented in **Figure 2C** to compare active lever presses during cue-primed reinstatement to those during the average of the last 3 days of extinction. Significant differences were found between extinction and cue-primed reinstatement for all treatment groups [Veh: *t*(1,7) = 4.036, *p* < 0.01; NAC60: *t*(1,8) = 4.302, *p* < 0.01; NAC30: *t*(1,9) = 3.651, *p* < 0.05], indicating that neither dose of NAC was able to prevent cue-primed reinstatement of alcohol-seeking.

### The Effects of CEF and CEFAZ on Cue-Primed Reinstatement of Alcohol-Seeking (Experiment 2)

**FIGURE 3:**
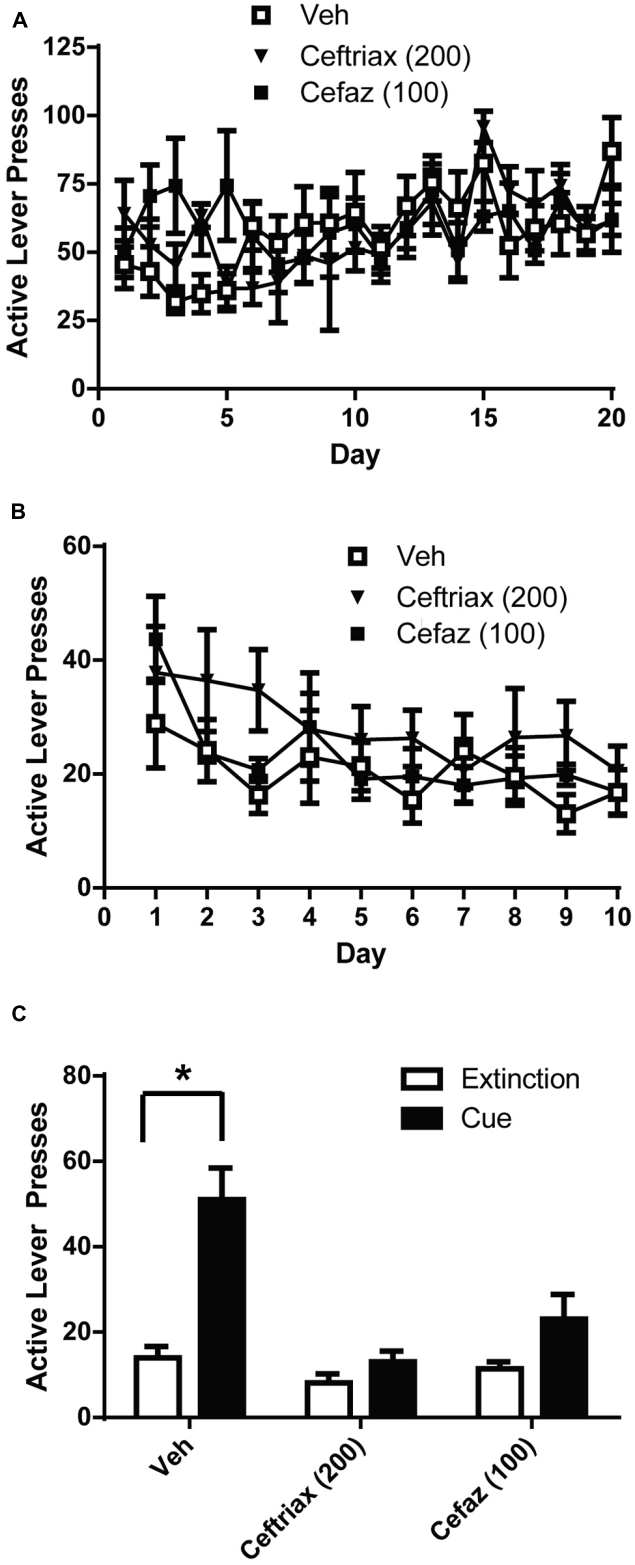
Ceftriaxone and cefazolin attenuated the reinstatement alcohol-seeking.** (A)** Mean active lever presses during the FR3 portion of the experiment did not differ between groups. **(B)** Mean active lever presses during extinction training did not differ between groups. **(C)** Only vehicle-treated animals reinstated lever-pressing during a cue-primed reinstatement test. *p < 0.05 comparing extinction to test.

Vehicle, CEF, and CEFAZ groups did not differ in the amount of active lever presses during self-administration (**Figure 3A**). A two-way ANOVA with repeated measures on Day was computed on the active lever presses during alcohol self-administration (**Figure 3A**) and revealed a significant effect of Day [*F*(19,380) = 2.612, *p* < 0.01], but not Group [*F*(2,20) = 0.370, n.s.]. There was not a significant Day × Group interaction [*F*(19,380) = 1.277, n.s.]. Similarly, a two-way ANOVA was computed on the number of lever presses during extinction training (**Figure 3B**) and revealed a significant effect of Day [*F*(9,180) = 8.050, *p* < 0.001], but not Group [*F*(2,20) = 0.989, n.s.]. There was not a significant Day × Group interaction [*F*(2,180) = 1.249, n.s.]. We used paired-sample *t*-tests to compare active lever presses during cue-primed reinstatement to those during the last 3 days of extinction (**Figure 3C**). A significant difference between extinction and cue-primed reinstatement was only observed for vehicle-treated animals [*t*(1,8) = 2.892, *p* < 0.05], indicating that CEF [*t*(1,6) = 1.3998, n.s.] and CEFAZ [*t*(1,6) = 2.090, n.s.] attenuated cue-primed reinstatement of alcohol-seeking.

### CEF and CEFAZ Did Not Alter Food and Sucrose Self-Administration or Weight (Experiment 3)

**FIGURE 4:**
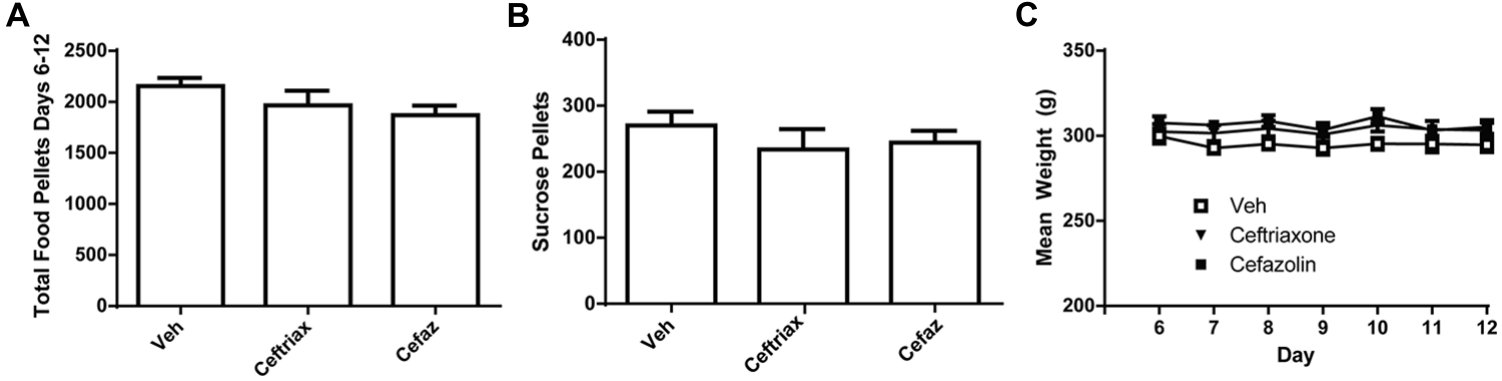
Ceftriaxone and cefazolin did not alter the motivation to self-administer regular chow or sucrose pellets. **(A)** CEF and CEFAZ did not alter the self-administration of regular chow pellets. **(B)** CEF and CEFAZ did not alter the mean number of sucrose pellets self-administered. **(C)** Mean body weight did not differ between vehicle-, CEF- or CEFAZ-treated rats.

Because CEF and CEFAZ treatment commenced following the session on Day 5, statistical analyses were conducted only on the data during the period of antibiotic (or vehicle) treatment (Days 6–12). One animal was excluded from data analysis because his lever pressing on Day 13 was greater than two standard deviations from the mean value. A one-ANOVA was computed on active lever presses during self-administration and revealed no effect of treatment group [*F*(2,17) = 2.128, n.s.; **Figure 4A**]. We also conducted a two-way ANOVA with repeated measures on Day and this analysis revealed no significant effects [Day: *F*(6,102) = 1.963, n.s.; Day × Group: *F*(12,102) = 1.375, n.s.; Group: *F*(2,17) = 2.361, n.s.]. A one-way ANOVA was used to determine if there were group differences in the number of sucrose pellets earned on Day 13 of the experiment (**Figure 4B**) and found no effect [*F*(2,17) = 1.068, n.s.]. There was also no difference in weight over time between the three treatment groups (**Figure 4C**). A two-way RM ANOVA found an effect of Day [*F*(11,102) = 9.145, *p* < 0.001] but no Day × Group interaction [*F*(12,102) = 2.507, n.s.] or effect of Group [*F*(2,17) = 1.924, n.s.] on animal weight.

### CEFAZ Did Not Attenuate the Reinstatement of Cocaine-Seeking (Experiment 4)

**FIGURE 5:**
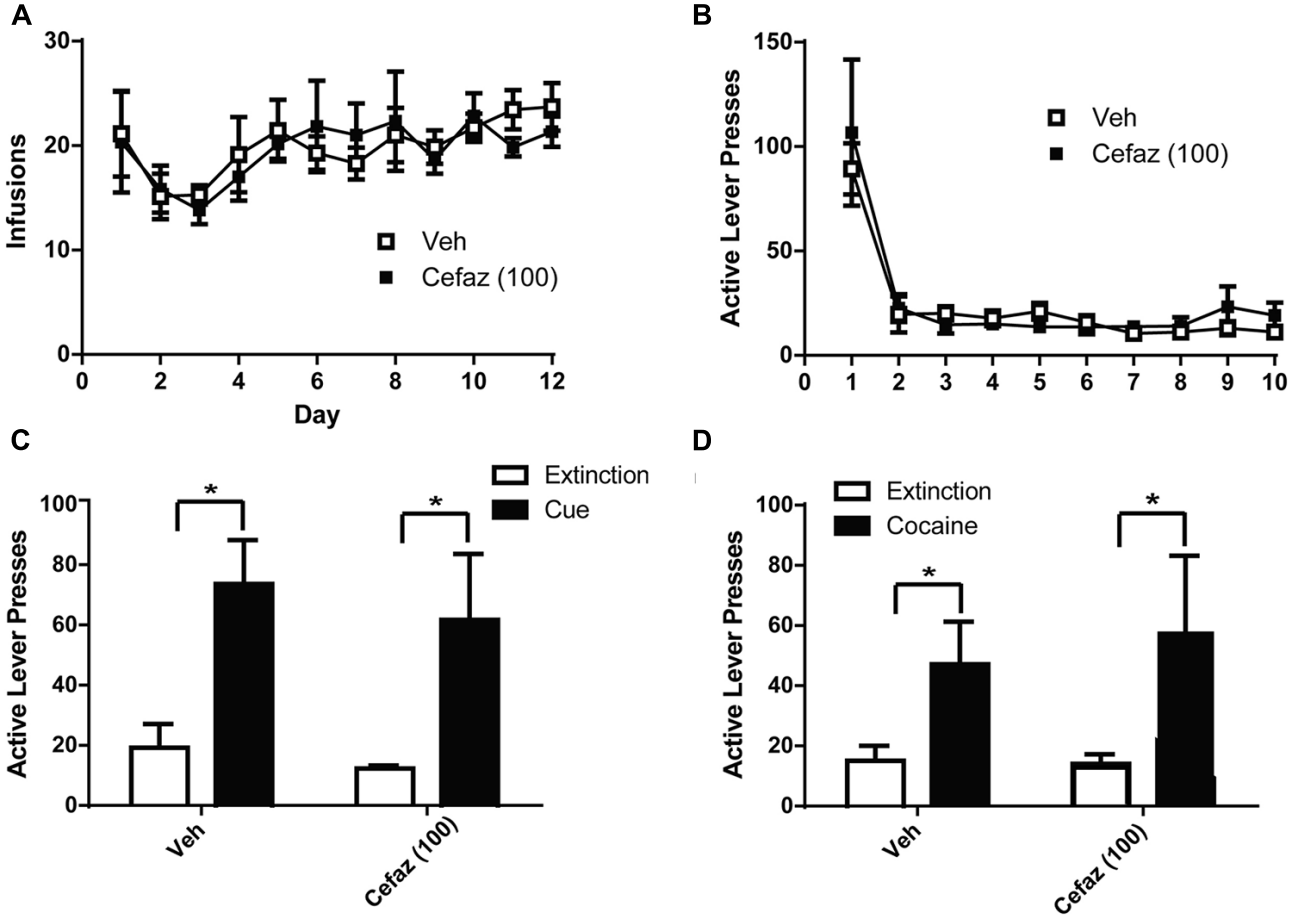
Cefazolin did not attenuate cue- or cocaine-primed reinstatement of cocaine-seeking. **(A)** Animals later treated with vehicle or CEFAZ did not differ in the number of infusions attained during cocaine self-administration. **(B)** Vehicle- and CEFAZ-treated animals did not differ in active lever presses during extinction training. **(C)** CEFAZ did not attenuate cue-primed reinstatement. **(D)** CEFAZ did not attenuate cocaine-primed reinstatement. *p < 0.05 comparing extinction to test.

A two-way ANOVA computed on the amount of cocaine intake (**Figure 5A**) revealed no group differences [*F*(1,12) = 0.357, n.s.], no effect of Day [*F*(11,132) = 1.562, n.s.], and no Day × Group interaction [*F*(12,132) = 0.453, n.s.]. There were also no group differences in extinction training [*F*(1,12) = 0.472, n.s.] and no Day × Group interaction [*F*(12,108) = 0.696, n.s.] but there was an effect of Day [*F*(9,108) = 19.076, *p* < 0.001] as both groups decreased lever pressing during the course of training (**Figure 5B**). Paired-sample *t*-tests revealed significant differences between extinction and cue-primed reinstatement for both CEFAZ [*t*(1,5) = 2.834, *p* < 0.05] and vehicle-treated [*t*(1,7) = 4.453, *p* < 0.01] animals (**Figure 5C**). Paired-sample *t*-tests revealed significant differences between extinction and cocaine-primed reinstatement for both CEFAZ [*t*(1,5) = 2.834, *p* < 0.05] and vehicle-treated [*t*(1,7) = 2.614, *p* < 0.05] animals (**Figure 5D**). Thus, CEFAZ did not attenuate cue- or cocaine-primed reinstatement. Furthermore, an independent sample *t*-test showed no lever pressing differences between vehicle- and CEFAZ-treated groups during the cue [*t*(1,12) = 0.272, n.s.] and cocaine tests[*t*(1,12) = 0.269, n.s.].

## Discussion

Here, we demonstrated that training Sprague-Dawley rodents to self-administer unsweetened alcohol in the operant chamber can be accomplished using daily training sessions without lengthy (14 h; e.g., Simms et al., 2010) operant sessions on alternating days. This is the first demonstration of such an effect in the Sprague-Dawley strain. Augier et al. (2014) recently demonstrated the same effect in Wistar rats. Using the procedure outlined here (**Figure 1A**) reduced the time to complete the experiment by 4 weeks relative to a procedure utilizing intermittent (MWF) operant sessions (e.g., Simms et al., 2010). This represents a significant conservation of lab resources.

We used this operant procedure to investigate the ability of NAC, CEF, and CEFAZ to attenuate cue-primed reinstatement. Five days of NAC treatment (30 or 60 mg/kg) did not attenuate cue-primed reinstatement (**Figure 2C**). We found that chronic CEF (200 mg/kg) and CEFAZ (100 mg/kg) attenuated cue-primed reinstatement of alcohol-seeking (**Figure 3C**). Both NAC and CEF attenuate cocaine and heroin reinstatement (Baker et al., 2003; Zhou and Kalivas, 2008; Knackstedt et al., 2010; Reichel et al., 2011; Sondheimer and Knackstedt, 2011; Shen et al., 2014). Furthermore, both compounds have been documented to similarly restore glutamate homeostasis in the NAc following cocaine (see **Table 1**). Both compounds increase GLT-1 and system x_c_-/xCT expression and function (Knackstedt et al., 2010; Trantham-Davidson et al., 2012), increase basal glutamate levels (Baker et al., 2003; Trantham-Davidson et al., 2012), and normalize potentiated glutamate synapses (Moussawi et al., 2011; Trantham-Davidson et al., 2012). Because CEF and NAC modulate post-cocaine glutamate homeostasis in similar manner, and the present results reveal a dissociation between the ability of the compounds to attenuate alcohol reinstatement, CEF and CEFAZ likely attenuate alcohol reinstatement via a glutamate-independent mechanism.

**Table 1:** Summary of *N*-acetylcysteine and ceftriaxone effects on cocaine-induced adaptations in the nucleus accumbens.

Post-cocaine adaptation	Compound	
	*N*-acetylcysteine	Ceftriaxone
Restore (elevate) basal glutamate	Yes Baker et al. (2003)	Yes Trantham-Davidson et al. (2012)
Prevent glutamate release during cocaine relapse	Yes Baker et al. (2003)	Yes Trantham-Davidson et al. (2012)
Increase GLT-1 expression/function	Yes (expression) Knackstedt et al. (2010) function not assessed	Yes Knackstedt et al. (2010), Trantham-Davidson et al. (2012)
Increase xCT expression/system xc- function	Yes Baker et al. (2003), Knackstedt et al. (2010)	Yes Knackstedt et al. (2010), Trantham-Davidson et al. (2012)
Stimulate mGluR2/3 function	Yes Moussawi et al. (2011)	Not yet assessed
Normalize potentiated accumbens synapses	Yes Moussawi et al. (2011)	Yes Trantham-Davidson et al. (2012)

Cefazolin has been shown to reduce GABA_A_ transmission (Yamazaki et al., 2002; Sugimoto et al., 2003). Positive allosteric modulation of GABA_A_ enhances reinstatement of alcohol-seeking (Finn et al., 2008; Ramaker et al., 2014) and thus a reduction in GABA transmission by CEFAZ may be the mechanism of action by which it reduces alcohol reinstatement. CEF has not been fully evaluated for its ability to alter GABA transmission although one report exists showing that it does not alter GABA_A_ receptor binding in the hippocampus or frontal and parietal cortices (Inui et al., 2013). CEFAZ has not been demonstrated to alter glutamate transmission: it does not alter NMDA function (Yamazaki et al., 2002; Sugimoto et al., 2003) and was not determined to potently induce GLT-1 expression as do CEF and penicillin (Rothstein et al., 2005). The data presented in **Figures 5C, D** provide evidence that CEFAZ does not alter cue- or cocaine-primed reinstatement of cocaine-seeking. Thus, the mechanism by which CEFAZ attenuates alcohol reinstatement likely does not involve modulation of glutamate neurotransmission in the NAs.

Ceftriaxone and CEFAZ are antibiotics and have the potential to produce gastrointestinal distress upon chronic administration. Oral alcohol self-administration in rodents and even alcohol-seeking during cue-primed reinstatements tests may be impacted by this side-effect. Thus we sought to determine the effects of these antibiotics on the operant self-administration of regular chow and sucrose pellets. We found no effect of either antibiotic on the self-administration of chow (**Figure 4A**) or sucrose (**Figure 4B**) pellets or on body weight during the course of CEF and CEFAZ injections (**Figure 4C**). These findings are in agreement with those of Ward et al. (2011) who found no effect of CEF on the self-administration of sweet food in mice. Interestingly, the chronic administration of NAC, which did not attenuate alcohol-seeking here, has been demonstrated to reduce operant self-administration of regular chow (Ramirez-Niño et al., 2013). Thus, we can conclude that the ability of CEF and CEFAZ to attenuate cue-primed alcohol-seeking is likely not dependent on any gastrointestinal side-effects of these compounds.

Because CEFAZ had no impact on cocaine reinstatement (**Figure 5C**), it may not be capable of restoring glutamate homeostasis after cocaine. Thus, this is not likely the mechanism by which CEF and CEFAZ attenuate cue-primed reinstatement of alcohol-seeking. In support of this, NAC, which does alter glutamate homeostasis, did not attenuate cue-primed alcohol-seeking. This conclusion is surprising in light of the evidence supporting glutamate signaling in this behavior. In alcohol-preferring (P) rats, CEF reduces the consumption of alcohol acutely and after a period of withdrawal (Sari et al., 2013; Alhaddad et al., 2014). In P rats, the ability of CEF to modulate alcohol-seeking was accompanied by increases in GLT-1 and xCT (Sari et al., 2013; Alhaddad et al., 2014). These results indicate that the modulation of glutamate homeostasis may be the mechanism by which CEF attenuates alcohol consumption, at least in P rats. However, the increased expression of these proteins was not shown to be the mechanism by which CEF exerted its behavioral effects. An alternative explanation for the failure of NAC to attenuate alcohol reinstatement in the present manuscript is that the operationalization of the alcohol-seeking response may have altered the underlying neurobiology of alcohol-seeking, rendering the manipulation of GLT-1 and xCT (and thereby glutamate levels) ineffective at preventing reinstatement.

The lack of an effect of NAC on alcohol-seeking here does not discount the involvement of glutamate in alcohol reinstatement. Antagonism of the post-synaptic mGluR5 and AMPA receptors attenuates cue-induced reinstatement (Backstrom and Hyytia, 2004; Backstrom et al., 2004; Sanchis-Segura et al., 2006; Sinclair et al., 2012). Stimulating the pre-synaptic glutamate autoreceptor mGluR2/3 also attenuates cue-induced reinstatement of alcohol-seeking (Zhao et al., 2006), presumably by decreasing pre-synaptic glutamate release. While both cocaine and alcohol reinstatement are driven by synaptic glutamate release in the NA core during the reinstatement event (McFarland et al., 2003; Gass et al., 2011) and NAC and CEF attenuate that increase during *cocaine* reinstatement (Baker et al., 2003; Trantham-Davidson et al., 2012), NAC (and potentially CEF) may not be able to attenuate glutamate release during *alcohol* reinstatement. The reason for this may be that cocaine and alcohol have opposite effects on basal glutamate levels in the NA core. Cocaine decreases (Baker et al., 2003) while alcohol increases (Melendez et al., 2005; Griffin Iii et al., 2013) basal glutamate levels. Both NAC and CEF increase basal glutamate levels after cocaine but not in drug-naïve animals

(Baker et al., 2003; Trantham-Davidson et al., 2012). After NAC treatment, this increased basal glutamate derived from system x_c_- restores tone on mGluR2/3 autoreceptors (Moussawi et al., 2011), thereby restoring the ability of these receptors to exert negative feedback on glutamate release during reinstatement. It is possible that increased basal glutamate following alcohol also causes a failure in mGluR2/3 signaling that is insensitive to NAC treatment. The effects of CEF on mGluR2/3 function have not yet been investigated (see **Table Table 1**). Future experiments examining basal glutamate levels 2–3 weeks after cessation of alcohol self-administration in combination with NAC and CEF would provide valuable data regarding the potential value in manipulating basal glutamate to treat alcohol relapse.

Overall, these results indicate that training Sprague-Dawley rats to self-administer alcohol in the operant chamber can be accomplished without sucrose fading and by using daily training sessions without lengthy operant sessions. We found that modulating glutamate homeostasis likely will not represent a successful treatment strategy for preventing relapse to alcohol-seeking. Future studies should investigate GABA modulation as a potential mechanism of action for CEF and as a method of attenuating alcohol relapse.

## Conflict of Interest Statement

The Reviewer Jamie Peters declares that, despite being affiliated to the same institution as the author Ana Weiland, the review process was handled objectively and no conflict of interest exists. The authors declare that the research was conducted in the absence of any commercial or financial relationships that could be construed as a potential conflict of interest.

## References

[B1] Alhaddad H. Das S. C. Sari Y. (2014). Effects of CEF on alcohol intake: a possible role for xCT and GLT-1 isoforms modulation of glutamate levels in P rats. *Psychopharmacology* 231 4049–4057 10.1007/s00213-014-3545-y 24687412 PMC4176549

[B2] Amen S. L. Piacentine L. B. Ahmad M. E. Li S. J. Mantsch J. R. Risinger R. C. (2011). Repeated *N*-acetylcysteine reduces cocaine seeking in rodents and craving in cocaine-dependent humans. *Neuropsychopharmacology* 36 871–878 10.1038/npp.2010.226 21160464 PMC3052624

[B3] Augier E. Flanigan M. Dulman R. S. Pincus A. Schank J. R. Rice K. C. (2014). Wistar rats acquire and maintain self-administration of 20% ethanol without water deprivation, saccharin/sucrose fading, or extended access training. *Psychopharmacology* 231 4561–4568 10.1007/s00213-014-3605-3 24858375 PMC4988093

[B4] Avena N. M. Rada P. Hoebel B. G. (2008). Evidence for sugar addiction: behavioral and neurochemical effects of intermittent, excessive sugar intake. *Neurosci. Biobehav. Rev.* 32 20–39 10.1016/j.neubiorev.2007.04.019 17617461 PMC2235907

[B5] Backstrom P. Bachteler D. Koch S. Hyytia P. Spanagel R. (2004). mGluR5 antagonist MPEP reduces ethanol-seeking and relapse behavior. *Neuropsychopharmacology* 29 921–928 10.1038/sj.npp.1300381 14735132

[B6] Backstrom P. Hyytia P. (2004). Ionotropic glutamate receptor antagonists modulate cue-induced reinstatement of ethanol-seeking behavior. *Alcohol. Clin. Exp. Res.* 28 558–565 10.1097/01.ALC.0000122101.13164.21 15100606

[B7] Baker D. A. McFarland K. Lake R. W. Shen H. Tang X. C. Toda S. (2003). Neuroadaptations in cystine-glutamate exchange underlie cocaine relapse. *Nat. Neurosci.* 6 743–749 10.1038/nn1069 12778052

[B8] Baker D. A. Tran-Nguyen T. L. Fuchs R. A. Neisewander J. L. (2001). Influence of individual differences and chronic fluoxetine treatment on cocaine-seeking behavior in rats. *Psychopharmacology* 155 18–26 10.1007/s002130000676 11374332

[B9] Baker D. A. Xi Z. X. Shen H. Swanson C. J. Kalivas P. W. (2002). The origin and neuronal function of in vivo nonsynaptic glutamate. *J. Neurosci.* 22 9134–9141. 12388621 10.1523/JNEUROSCI.22-20-09134.2002 PMC6757683

[B10] Bongiovanni M. See R. E. (2008). A comparison of the effects of different operant training experiences and dietary restriction on the reinstatement of cocaine-seeking in rats. *Pharmacol. Biochem. Behav.* 89 227–233 10.1016/j.pbb.2007.12.019 18230406 PMC2267375

[B11] Carrillo J. Howard E. C. Moten M. Houck B. D. Czachowski C. L. Gonzales R. A. (2008). A 3-day exposure to 10% ethanol with 10% sucrose successfully initiates ethanol self-administration. *Alcohol* 42 171–178 10.1016/j.alcohol.2008.01.005 18420112 PMC2577812

[B12] Colantuoni C. Rada P. McCarthy J. Patten C. Avena N. M. Chadeayne A. (2002). Evidence that intermittent, excessive sugar intake causes endogenous opioid dependence. *Obes. Res.* 10 478–488 10.1038/oby.2002.66 12055324

[B13] Deroche V. Le Moal M. Piazza P. V. (1999). Cocaine self-administration increases the incentive motivational properties of the drug in rats. *Eur. J. Neurosci.* 11 2731–2736 10.1046/j.1460-9568.1999.00696.x 10457169

[B14] Epstein D. H. Preston K. L. Stewart J. Shaham Y. (2006). Toward a model of drug relapse: an assessment of the validity of the reinstatement procedure. *Psychopharmacology (Berl)* 189 1–16 10.1007/s00213-006-0529-6 17019567 PMC1618790

[B15] Finn D. A. Mark G. P. Fretwell A. M. Gililland-Kaufman K. R. Strong M. N. Ford M. M. (2008). Reinstatement of ethanol and sucrose seeking by the neurosteroid allopregnanolone in C57BL/6 mice. *Psychopharmacology* 201 423–433 10.1007/s00213-008-1303-8 18758755 PMC4767154

[B16] Gass J. T. Sinclair C. M. Cleva R. M. Widholm J. J. Olive M. F. (2011). Alcohol-seeking behavior is associated with increased glutamate transmission in basolateral amygdala and nucleus accumbens as measured by glutamate-oxidase-coated biosensors. *Addict. Biol.* 16 215–228 10.1111/j.1369-1600.2010.00262.x 21054692 PMC3058760

[B17] Griffin W. C. III. Haun H. L. Hazelbaker C. L. Ramachandra V. S. Becker H. C. (2013). Increased extracellular glutamate in the nucleus accumbens promotes excessive ethanol drinking in ethanol dependent mice. *Neuropsychopharmacology* 39 707–717 10.1038/npp.2013.256 24067300 PMC3895249

[B18] Heilig M. Egli M. (2006). Pharmacological treatment of alcohol dependence; target symptoms and target mechanisms. *Pharmacol. Ther.* 111 855–876 10.1016/j.pharmthera.2006.02.001 16545872

[B19] Inui T. Alessandri B. Heimann A. Nishimura F. Frauenknecht K. Sommer C. (2013). Neuroprotective effect of CEF on the penumbra in a rat venous ischemia model. *Neuroscience* 242 1–10 10.1016/j.neuroscience.2013.03.018 23523747

[B20] Janak P. H. Michael Gill T. (2003). Comparison of the effects of allopregnanolone with direct GABAergic agonists on ethanol self-administration with and without concurrently available sucrose. *Alcohol* 30 1–7 10.1016/S0741-8329(03)00068-5 12878269

[B21] Kalivas P. W. Lalumiere R. T. Knackstedt L. Shen H. (2009). Glutamate transmission in addiction. *Neuropharmacology* 56(Suppl. 1) 169–173 10.1016/j.neuropharm.2008.07.011 18675832 PMC3280337

[B22] Kenna G. A. McGeary J. E. Swift R. M. (2004). Pharmacotherapy, pharmacogenomics, and the future of alcohol dependence treatment, Part 2. *Am. J. Health Syst. Pharm.* 61 2380–2388. 15581261 10.1093/ajhp/61.22.2380

[B23] Knackstedt L. A. Kalivas P. W. (2009). Glutamate and reinstatement. *Curr. Opin. Pharmacol.* 9 59–64 10.1016/j.coph.2008.12.003 19157986 PMC2667702

[B24] Knackstedt L. A. Melendez R. I. Kalivas P. W. (2010). CEF restores glutamate homeostasis and prevents relapse to cocaine seeking. *Biol. Psychiatry* 67 81–84 10.1016/j.biopsych.2009.07.018 19717140 PMC2795043

[B25] Knackstedt L. A. Trantham-Davidson H. Schwendt M. (2014). The role of ventral and dorsal striatum mGluR5 in relapse to cocaine-seeking and extinction learning. *Addict. Biol.* 19 87–101 10.1111/adb.12061 23710649 PMC3762937

[B26] Koob G. F. Weiss F. (1990). Pharmacology of drug self-administration. *Alcohol* 7 193–197 10.1016/0741-8329(90)90004-V 1970479

[B27] Lee S. G. Su Z. Z. Emdad L. Gupta P. Sarkar D. Borjabad A. (2008). Mechanism of ceftriaxone induction of excitatory amino acid transporter-2 expression and glutamate uptake in primary human astrocytes. *J. Biol. Chem.* 283 13116–3123 10.1074/jbc.M707697200 18326497 PMC2442320

[B28] Lewerenz J. Albrecht P. Tien M. L. Henke N. Karumbayaram S. Kornblum H. I. (2009). Induction of Nrf2 and xCT are involved in the action of the neuroprotective antibiotic ceftriaxone in vitro. *J. Neurochem.* 111 332–343 10.1111/j.1471-4159.2009.06347.x 19694903

[B29] McBride W. J. Li T. K. (1998). Animal models of alcoholism: neurobiology of high alcohol-drinking behavior in rodents. *Crit. Rev. Neurobiol.* 12 339–369 10.1615/CritRevNeurobiol.v12.i4.40 10348615

[B30] McFarland K. Lapish C. C. Kalivas P. W. (2003). Prefrontal glutamate release into the core of the nucleus accumbens mediates cocaine-induced reinstatement of drug-seeking behavior. *J. Neurosci.* 23 3531–3537. 12716962 10.1523/JNEUROSCI.23-08-03531.2003 PMC6742291

[B31] Meisch R. A. Thompson T. (1972). Ethanol intake during schedule-induced polydipsia. *Physiol. Behav.* 8 471–475 10.1016/0031-9384(72)90331-9 5037545

[B32] Melendez R. I. Hicks M. P. Cagle S. S. Kalivas P. W. (2005). Ethanol exposure decreases glutamate uptake in the nucleus accumbens. *Alcohol. Clin. Exp. Res.* 29 326–333 10.1097/01.ALC.0000156086.65665.4D 15770106

[B33] Moussawi K. Pacchioni A. Moran M. Olive M. F. Gass J. T. Lavin A. (2009). N-Acetylcysteine reverses cocaine-induced metaplasticity. *Nat. Neurosci.* 12 182–189 10.1038/nn.2250 19136971 PMC2661026

[B34] Moussawi K. Zhou W. Shen H. Reichel C. M. See R. E. Carr D. B. (2011). Reversing cocaine-induced synaptic potentiation provides enduring protection from relapse. *Proc. Natl. Acad. Sci. U.S.A.* 108 385–390 10.1073/pnas.1011265108 21173236 PMC3017187

[B35] Nie H. Janak P. H. (2003). Comparison of reinstatement of ethanol- and sucrose-seeking by conditioned stimuli and priming injections of allopregnanolone after extinction in rats. *Psychopharmacology* 168 222–228 10.1007/s00213-003-1468-0 12719962

[B36] NIH. (2006). “National epidemiologic survey on alcohol and related conditions: 2001–2002. Alcohol use and alcohol use disorders in the United States," in *Alcohol Epidemiologic Data Reference Manual* Vol. 8 (Bethesda, MD: National Institute on Alcohol Abuse and Alcoholism).

[B37] Pinel J. P. Huang E. (1976). Effects of periodic withdrawal on ethanol and saccharin selection in rats. *Physiol. Behav.* 16 693–698 10.1016/0031-9384(76)90238-9 981364

[B38] Ramaker M. J. Ford M. M. Phillips T. J. Finn D. A. (2014). Differences in the reinstatement of ethanol seeking with ganaxolone and gaboxadol. *Neuroscience* 272 180–187 10.1016/j.neuroscience.2014.04.065 24814021 PMC4122668

[B39] Ramirez-Niño A. M. D’Souza M. S. Markou A. (2013). N-acetylcysteine decreased nicotine self-administration and cue-induced reinstatement of nicotine seeking in rats: comparison with the effects of N-acetylcysteine on food responding and food seeking. *Psychopharmacology* 225 473–482 10.1007/s00213-012-2837-3 22903390 PMC3697766

[B40] Reichel C. M. Moussawi K. Do P. H. Kalivas P. W. See R. E. (2011). Chronic N-acetylcysteine during abstinence or extinction after cocaine self-administration produces enduring reductions in drug seeking. *J. Pharmacol. Exp. Ther.* 337 487–493 10.1124/jpet.111.179317 21303920 PMC3083102

[B41] Rothstein J. D. Patel S. Regan M. R. Haenggeli C. Huang Y. H. Bergles D. E. (2005). Beta-lactam antibiotics offer neuroprotection by increasing glutamate transporter expression. *Nature* 433 73–77 10.1038/nature03180 15635412

[B42] Samson H. H. (1986). Initiation of ethanol reinforcement using a sucrose-substitution procedure in food- and water-sated rats. *Alcohol. Clin. Exp. Res.* 10 436–442 10.1111/j.1530-0277.1986.tb05120.x 3530023

[B43] Samson H. H. Files F. J. Denning C. Marvin S. (1998). Comparison of ethanol-preferring and nonpreferring selectively bred rat lines. I. Ethanol initiation and limited access operant self-administration. *Alcohol. Clin. Exp. Res.* 22 2133–2146 10.1111/j.1530-0277.1998.tb05927.x 9884162

[B44] Samson H. H. Sharpe A. L. Denning C. (1999). Initiation of ethanol self-administration in the rat using sucrose substitution in a sipper-tube procedure. *Psychopharmacology* 147 274–279 10.1007/s002130051167 10639685

[B45] Samson H. H. Tolliver G. A. Pfeffer A. O. Sadeghi K. G. Mills F. G. (1987). Oral ethanol reinforcement in the rat: effect of the partial inverse benzodiazepine agonist RO15-4513. *Pharmacol. Biochem. Behav.* 27 517–519 10.1016/0091-3057(87)90357-1 3659074

[B46] Sanchis-Segura C. Borchardt T. Vengeliene V. Zghoul T. Bachteler D. Gass P. (2006). Involvement of the AMPA receptor GluR-C subunit in alcohol-seeking behavior and relapse. *J. Neurosci.* 26 1231–1238 10.1523/JNEUROSCI.4237-05.2006 16436610 PMC6674564

[B47] Sari Y. Sreemantula S. N. Lee M. R. Choi D. S. (2013). CEF treatment affects the levels of GLT1 and ENT1 as well as ethanol intake in alcohol-preferring rats. *J. Mol. Neurosci.* 51 779–787 10.1007/s12031-013-0064-y 23893122 PMC3797852

[B48] Shen H. W. Scofield M. D. Boger H. Hensley M. Kalivas P. W. (2014). Synaptic glutamate spillover due to impaired glutamate uptake mediates heroin relapse. *J. Neurosci.* 34 5649–5657 10.1523/JNEUROSCI.4564-13.2014 24741055 PMC3988415

[B49] Simms J. A. Bito-Onon J. J. Chatterjee S. Bartlett S. E. (2010). Long-Evans rats acquire operant self-administration of 20% ethanol without sucrose fading. *Neuropsychopharmacology* 35 1453–1463 10.1038/npp.2010.15 20200505 PMC2869399

[B50] Simms J. A. Steensland P. Medina B. Abernathy K. E. Chandler L. J. Wise R. (2008). Intermittent access to 20% ethanol induces high ethanol consumption in Long-Evans and Wistar rats. *Alcohol. Clin. Exp. Res.* 32 1816–1823 10.1111/j.1530-0277.2008.00753.x 18671810 PMC3151464

[B51] Sinclair C. M. Cleva R. M. Hood L. E. Olive M. F. Gass J. T. (2012). mGluR5 receptors in the basolateral amygdala and nucleus accumbens regulate cue-induced reinstatement of ethanol-seeking behavior. *Pharmacol. Biochem. Behav.* 101 329–335 10.1016/j.pbb.2012.01.014 22296815 PMC3310253

[B52] Sondheimer I. Knackstedt L. A. (2011). CEF prevents the induction of cocaine sensitization and produces enduring attenuation of cue- and cocaine-primed reinstatement of cocaine-seeking. *Behav. Brain Res.* 225 252–258 10.1016/j.bbr.2011.07.041 21824497 PMC3170490

[B53] Soyka M. Roesner S. (2006). New pharmacological approaches for the treatment of alcoholism. *Expert Opin. Pharmacother.* 7 2341–2353 10.1517/14656566.7.17.2341 17109610

[B54] Sugimoto M. Uchida I. Mashimo T. Yamazaki S. Hatano K. Ikeda F. (2003). Evidence for the involvement of GABA(A) receptor blockade in convulsions induced by cephalosporins. *Neuropharmacology* 45 304–314 10.1016/S0028-3908(03)00188-6 12871648

[B55] Trantham-Davidson H. LaLumiere R. T. Reissner K. J. Kalivas P. W. Knackstedt L. A. (2012). CEF normalizes nucleus accumbens synaptic transmission, glutamate transport, and export following cocaine self-administration and extinction training. *J. Neurosci.* 32 12406–12410 10.1523/JNEUROSCI.1976-12.2012 22956831 PMC3465971

[B56] Vacca G. Serra S. Brunetti G. Carai M. A. Samson H. H. Gessa G. L. (2002). Operant self-administration of ethanol in sardinian alcohol-preferring rats. *Alcohol. Clin. Exp. Res.* 26 1678–1685 10.1111/j.1530-0277.2002.tb02470.x 12436056

[B57] Walker B. M. Koob G. F. (2007). The gamma-aminobutyric acid-B receptor agonist baclofen attenuates responding for ethanol in ethanol-dependent rats. *Alcohol. Clin. Exp. Res.* 31 11–18 10.1111/j.1530-0277.2006.00259.x 17207096 PMC2768469

[B58] Walker B. M. Rasmussen D. D. Raskind M. A. Koob G. F. (2008). Alpha 1-noradrenergic receptor antagonism blocks dependence-induced increases in responding for ethanol. *Alcohol* 42 91–97 10.1016/j.alcohol.2007.12.002 18358987 PMC2587143

[B59] Ward S. J. Rasmussen B. A. Corley G. Henry C. Kim J. K. Walker E. A. (2011). Beta-lactam antibiotic decreases acquisition of and motivation to respond for cocaine, but not sweet food, in C57Bl/6 mice. *Behav. Pharmacol.* 22 370–373 10.1097/FBP.0b013e3283473c10 21543969 PMC3135779

[B60] Wayner M. J. Greenberg I. (1972). Effects of hypothalamic stimulation, acclimation and periodic withdrawal on ethanol consumption. *Physiol. Behav.* 9 737–740 10.1016/0031-9384(72)90043-1 4570173

[B61] Wise R. A. (1973). Voluntary ethanol intake in rats following exposure to ethanol on various schedules. *Psychopharmacologia* 29 203–210 10.1007/BF00414034 4702273

[B62] Yamazaki S. Mochizuki Y. Terai T. Sugimoto M. Uchida I. Matsuoka N. (2002). Intracerebroventricular injection of the antibiotic cefoselis produces convulsion in mice via inhibition of GABA receptors. *Pharmacol. Biochem. Behav.* 74 53–59 10.1016/S0091-3057(02)00947-4 12376152

[B63] Zhao Y. Dayas C. V. Aujla H. Baptista M. A. Martin-Fardon R. Weiss F. (2006). Activation of group II metabotropic glutamate receptors attenuates both stress and cue-induced ethanol-seeking and modulates c-fos expression in the hippocampus and amygdala. *J. Neurosci.* 26 9967–9974 10.1523/JNEUROSCI.2384-06.2006 17005860 PMC6674480

[B64] Zhou W. Kalivas P. W. (2008). N-acetylcysteine reduces extinction responding and induces enduring reductions in cue- and heroin-induced drug-seeking. *Biol. Psychiatry* 63 338–340 10.1016/j.biopsych.2007.06.008 17719565 PMC2709691

